# Solitary splenic metastasis from ovarian carcinosarcoma: a case report

**DOI:** 10.1186/1752-1947-5-56

**Published:** 2011-02-10

**Authors:** Alex B Olsen, Sabine Pargman, Thomas Gillespie

**Affiliations:** 1 Department of General Surgery, St. Joseph's Hospital and Medical Center, 350 W. Thomas Road, Phoenix, AZ 85013, USA; 2 Department of Pathology, St. Joseph's Hospital and Medical Center, 350 W. Thomas Road, Phoenix, AZ 85013, USA

## Abstract

**Introduction:**

Metastatic tumors to the spleen are rare but are usually found in conjunction with metastasis to other organs. The most common sources of splenic metastasis are breast, lung and colorectal cancers as well as melanoma and ovarian carcinoma. A solitary carcinosarcoma metastasis to the spleen of any origin is very rare. To the best of our knowledge, there are fewer than 30 reported cases of ovarian primary tumors with solitary metastasis to the spleen, and only three solitary primary carcinosarcomas to the spleen have been reported, of which one is female. We present what is, to the best of our knowledge, the first case of a solitary metastatic carcinosarcoma to the spleen arising from a primary ovarian carcinsarcoma.

**Case presentation:**

A 72-year-old Hispanic woman status post-total abdominal hysterectomy for ovarian carcinosarcoma presented with complaints of early satiety and abdominal pain for the past two months with a 30-lb unintentional weight loss. An initial computed tomographic scan of her abdomen and pelvis revealed a 30 cm × 27 cm splenic mass with displacement of the left kidney, stomach and liver. The patient was found to have a solitary metastatic carcinosarcoma of the spleen with biphasic epithelial (carcinomatous) and mesenchymal (sarcomatous) elements consistent with carcinosarcoma.

**Conclusion:**

Carcinosarcoma of the spleen is a rare tumor. Carcinosarcomas are a biphasic neoplasm comprising malignant epithelial and mesenchymal components arising from a stem cell capable of differentiation. They can arise anywhere in the female genital tract, most commonly from the endometrium. Even though it is rare, carcinosarcomas can metastasize to the spleen. This unique case of a solitary splenic metastasis from ovarian carcinosarcoma has particular interest in medicine, especially for the specialties of surgical oncology, pathology and hematology/oncology.

## Introduction

The most common malignancy arising from the spleen is lymphoreticular. Malignant nonlymphoreticular neoplasms involving the spleen are rare. Splenic metastases from solid tumors occur in late stages of a disease process, are due to hematogenous dissemination confined to the splenic parenchyma and are usually found in conjunction with metastases to other organs [1]. The most common primary sources include breast, lung, stomach, colorectal and ovarian cancers and melanoma [1,2]. Solitary splenic metastases have been documented in 93 patients. In regard to ovarian tumors, there are fewer than 30 cases of solitary parenchymal metastases reported in the literature. The most common histologic type seen in these patients is serous cystadenocarcinoma [3]. Carcinosarcoma is typically an extremely aggressive neoplasm histologically comprising epithelial (carcinomatous) and mesenchymal (sarcomatous) elements. Westra *et al*. [4] were the first to report a solitary primary carcinosarcoma to the spleen in a woman. The other two cases of primary carcinosarcomas of the spleen were reported in men [5,6]. We present a case of a metachronous solitary splenic metastasis from ovarian carcinosarcoma.

## Case presentation

**Figure 1:**
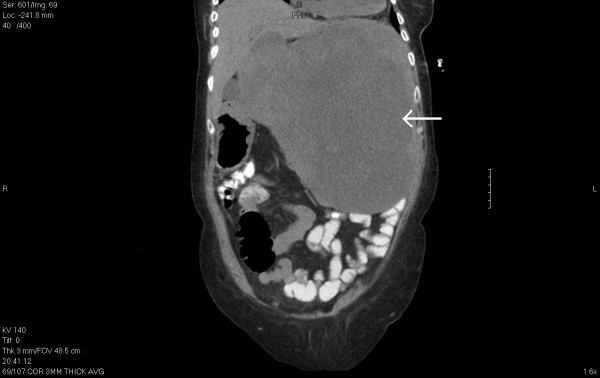
**A computed tomographic scan of a large splenic mass (coronal view)**. See attached arrow.

A 72-year-old Hispanic woman presented with complaints of early satiety and abdominal pain for the past two months with a 30-lb unintentional weight loss. Her medical history included a total abdominal hysterectomy with bilateral salpingo-oophorectomy performed in Mexico for ovarian cancer seven years previously. The pathology report described an ovarian carcinosarcoma. Her physical examination revealed a large left upper quadrant mass which was tender to palpation. An initial computed tomographic scan of her abdomen and pelvis revealed a 30 cm × 27 cm splenic mass in coronal view with displacement of the left kidney, stomach and liver (see attached arrow in Figure 1). No other masses concerning metastasis were noted.

Prior to her splenectomy and after appropriate vaccination (Menomune^® ^{A/C/Y/W-135, Meningococcal Polysaccharide Vaccine, Groups A, C, Y and W-135 Combine, Manufacturer Sanofi Pasteur located in Swiftwater, Pennsylvania, United States of America}, ActHIB^® ^{ Haemophilus b Conjugate Vaccine Manufacturer located Sanofi Pasteur located in Swiftwater, Pennsylvania, United States of America} and Pneumovax 23^® ^{pneumococcal vaccine polyvalent, Manufacturer MERCK located in Whitehouse Station, New Jersey, United States of America}), the patient underwent embolization of her splenic artery as a cautious approach to prevent hemorrhage.

**Figure 2:**
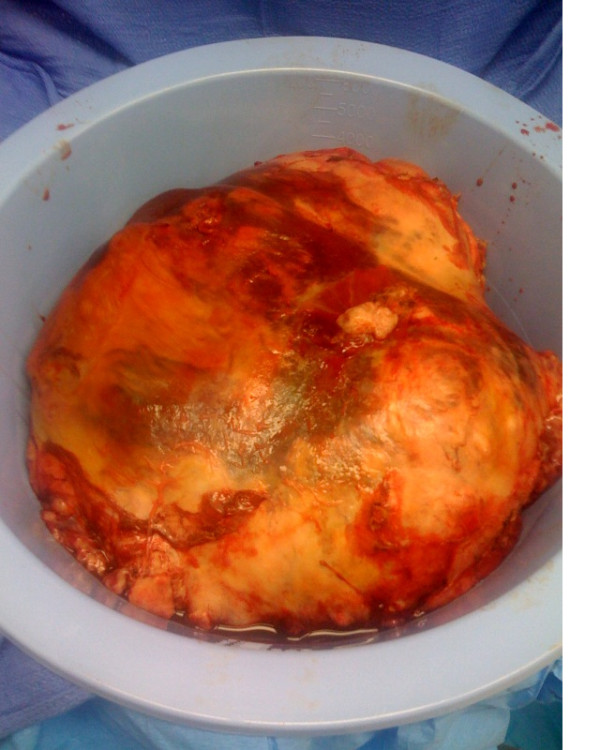
**Gross specimen measuring 30 cm × 27 cm × 20 cm (4.2 kg)**.

**Figure 3:**
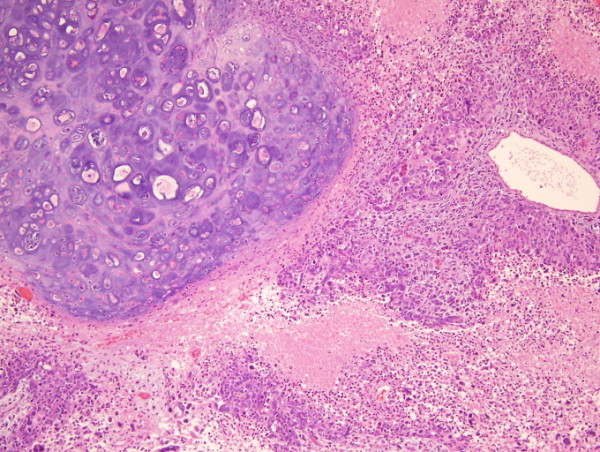
**Biphasic tumor with malignant stromal and malignant epithelial component, heterologous (original magnification, × 20; hematoxylin and eosin stain)**.

In addition to the planned splenectomy, a partial gastrectomy was performed because the splenic tumor directly invaded the proximal stomach. The splenic mass was delivered *en bloc *and without evidence of rupture. The specimen weighed 4.2 kg and had a grossly intact capsule beside the region in which it had invaded the stomach (Figure 2). The gross specimen measured 30 cm × 27 cm × 20 cm. Estimated blood loss was 2200 mL secondary to bleeding. A total of eight units of packed red blood cells, four units of fresh frozen plasma and two units of platelets were administered during the procedure. Post-operatively, the patient did well. She was extubated on post-operative day four. An upper gastrointestinal gastrograffin study showed no overt extraluminal contrast. The patient's symptoms of early satiety were relieved and her diet was advanced slowly. Her pathology demonstrated a carcinosarcoma with malignant stromal and epithelial components consistent with ovarian origin heterologous cartilage (original magnification, × 20; hematoxylin and eosin stain) (Figure 3).

## Discussion

The incidence of splenic metastases is between 2.3% and 7.1% [1]. Solitary metastasis to the spleen is secondary to hematogenous dissemination and is confined to the splenic parenchyma. The most common primary sources include breast, lung, stomach, ovarian and colorectal cancers and melanoma [1,2]. Skin melanoma has the highest rate of splenic metastases per primary tumor. More than 30% of patients with skin melanoma have splenic metastasis at autopsy [3]. Solitary splenic metastases have been reported in 93 cases. The time from the diagnosis of primary tumor to the discovery of solitary splenic metastasis ranges from zero to 264 months with a median of 28 months [7]. Colorectal and ovarian carcinomas are the most common sources of splenic metastases other than melanoma. In regard to ovarian carcinoma, the most prevalent type is serous cystadenocarcinoma, which has been reported in 17 cases [7].

Carcinosarcomas are tumors composed of both malignant epithelial and mesenchymal elements, and they arise primarily from the female reproductive tract. Carcinosarcomas with or without heterologous components typically have poorly differentiated endometrioid or serous histology and are often associated with *p53 gene *mutations [8]. Our patient's spleen showed a biphasic tumor composed of epithelial (carcinomatous) and mesenchymal (sarcomatous) elements consistent with carcinosarcoma. It is believed that the mesenchymal component differentiates from the epithelial component via a metaplastic process. The tumor did contain heterologous elements (that is, cartilage) in our case. Microscopic examination showed large strands and cords of anaplastic cells with pleomorphic nuclei. There was no involvement of the splenic capsule; however, the tumor was found to invade the serosa of the stomach with extension into the muscular propia with portions also involving the mucosa.

The pathogenesis of carcinosarcoma is poorly understood. Only two opposing theories have received strong support to explain the histological features found in this type of tumor. The monoclonal theory stipulates that these tumors are undifferentiated and have the capability to develop mesenchymal and epithelial components. On the other hand, the multiclonal theory supports the hypothesis that two separate cell lines fuse and differentiate into a single tumor [2,9]. It is important to distinguish a true carcinosarcoma from a collision tumor, in which a carcinoma and sarcoma arise in close proximity to one another. These tumors arise from different sources, forming a tumor border and never truly commingling [2,10]. Synchronous sarcoma and carcinoma arise from separate stem cells and merge in a biclonal process [9]. In our case, there was a single mass with mixed epithelial and mesenchymal components resembling a malignant Müllerian tumor, thus ruling out a collision tumor.

The pathogenesis of solitary splenic metastasis is unknown. The rarity of solitary splenic metastasis might be explained by (1) mechanical factors impeding hematogenous implantation of neoplastic cells because of the flow and course of blood through the splenic vasculature and (2) an inhibitory effect of the splenic microenvironment on neoplastic cells [1,7]. Other hypotheses that can explain the scarcity of splenic metastasis include the acute angle of the origin and tortuosity of the splenic artery, the role of the splenic capsule as a physical barrier, the lack of afferent lymphatics to the splenic metastasis and its rhythmic contractile properties [11].

Splenic metastases may occur by direct extension, transperitoneal spread, hematogenous route or lymphatogenous route or by a combination of these phenomena. The main metastatic pathway to the spleen is hematogenous, and it is generally accepted that by the time a metastatic tumor is found in the spleen, multiple secondary tumors can be found in other organs. Splenic metastasis is often associated with terminal cancer, and isolated splenic metastasis is rare [12]. Implantation of cancer cells in the splenic parenchyma may occur at the time of primary diagnosis but is clinically undetectable because the splenic microenvironment may not facilitate the growth of micrometastatic foci [3].

On the basis of gross and microscopic descriptions, splenic metastases can be categorized according by the type of splenic invasion: parenchymal-only, capsular or capsular with parenchymal invasion [11]. Ovarian cancer generally metastasizes via the lymphatic system or by peritoneal dissemination. As far as splenic metastasis of this malignancy is concerned, while disseminated lesions can sometimes be seen on the surface of the spleen, parenchymal splenic metastasis of ovarian cancer is relatively rare, and solitary metastasis is even rarer [13]. They can present as a solitary splenic mass synchronous or metachronous to the primary tumor. Our case is an example of a metachronous solitary splenic metastasis.

Splenic metastases are most often incidentally detected by ultrasonography or computed tomography in follow-up of cancer patients or in the work-up performed of any event related to cancer. Splenic metastases are a part of multi-visceral metastatic disease. Metastatic tumor of the spleen diagnosed in patients with widespread visceral dissemination of primary tumor has no clinical importance, because the overall prognosis is very poor. However, splenectomy can be performed as a palliative treatment option in those patients with symptomatic splenomegaly [7].

The appropriate choice of post-operative chemotherapy is debatable. Because of the rarity of this condition, no randomized trials have been conducted to determine the best therapy. The median overall survival in ovarian carcinosarcoma appears to be extended with the use of platinum-based therapies [14]. The recently published phase II Gynecologic Oncology Group (GOG) study [15] of single-agent cisplatin as initial chemotherapy in ovarian carcinosarcoma had 136 eligible patients enrolled between 1977 and 1996, underscoring the rarity of these tumors and the difficulty of enrollment of patients into prospective clinical trials. The GOG study showed an overall response rate of 20% with single-agent cisplatin, providing the first prospective evidence that platinum is active as an initial therapy for patients with carcinosarcoma of the ovary [15].

Duska *et al*. [16] reported on 28 patients (including two patients with recurrent disease) treated with platinum and taxane who had a total response rate of 72%. The overall median survival for the 28 patients was 27.1 months. Rutledge *et al*. [14] reported on 29 patients receiving adjuvant chemotherapy, with 11 patients receiving platinum and ifosfamide and 16 patients receiving platinum and a taxane. The overall survival was 21 months for the entire group, and the response rate was not detailed in the report. Though overall survival was improved with the use of ifosfamide and cisplatin for the entire cohort, there was no significant advantage for the advanced stage group, and multivariate analysis did not identify any statistically significant predictor of outcome [14].

## Conclusions

Carcinosarcomas are a biphasic neoplasm comprising malignant epithelial and mesenchymal components arising from a stem cell capable of differentiation. The most common sources of splenic metastasis are breast, lung, colorectal and ovarian carcinomas as well as melanoma. They can arise anywhere in the female genital tract, most commonly from the endometrium. It must be noted again that because of the rarity of this condition, there is no consensus on the appropriate chemotherapy regimen. Complete surgical resection appears to offer the best chance of long-term survival. Carcinosarcoma of the spleen is a rare tumor. Only three cases of solitary primary carcinosarcomas of the spleen have been reported. To the best of our knowledge, this is the first reported case of a metachronous solitary splenic metastasis from ovarian carcinosarcoma.

## Competing interests

The authors declare that they have no competing interests.

## Consent

Written informed consent was obtained from the patient for publication of this case report and the accompanying images. A copy of the written consent is available for review by the Editor-in-Chief of this journal.

## Authors' contributions

AO was involved in drafting the manuscript and acquisition of data. He analyzed and interpreted the patient data regarding the disease process. TG was the attending surgeon who made substantial contributions to the analysis and interpretation of data. He revised the manuscript critically for important intellectual content. SP performed the histological examination of the spleen and was a contributor in writing the manuscript. All authors read and approved the final manuscript.
